# Video Nasoendoscopic-Assisted Transoral Adenoidectomy with the PEAK PlasmaBlade: A Preliminary Report of a Case Series

**DOI:** 10.1155/2017/1536357

**Published:** 2017-03-28

**Authors:** Chao-Yin Kuo, Yuan-Yung Lin, Hsin-Chien Chen, Cheng-Ping Shih, Chih-Hung Wang

**Affiliations:** ^1^Department of Otolaryngology-Head and Neck Surgery, Tri-Service General Hospital, National Defense Medical Center, Taipei 11490, Taiwan; ^2^Graduate Institute of Medical Sciences, National Defense Medical Center, Taipei 11490, Taiwan; ^3^Graduate Institute of Microbiology and Immunology, National Defense Medical Center, Taipei 11490, Taiwan; ^4^Hualien Armed Forces General Hospital, Hualien County 97144, Taiwan

## Abstract

*Objectives*. The primary objective for this study is to evaluate the advantages, disadvantages, surgical applicability, and outcome of the pulsed electron avalanche knife (PEAK) PlasmaBlade in transoral adenoidectomy under direct visualization using video nasoendoscopy.* Patients and Methods*. In this series, six cases of adenoid hypertrophy showing varying clinical presentations in relation to its clinical course were surgically treated using a PEAK PlasmaBlade. Before and after surgery, all patients underwent nasal endoscopy to define the grading of hypertrophic adenoids and postoperative outcome. Pure tone audiometry and tympanometry tests were carried out to investigate the change in middle and inner ear functions.* Results*. The mean follow-up period was 23.8 months. Postoperatively, symptoms of otitis media with effusion were all relieved with closure of the air-bone gap (6/6). Other relevant ear complaints like tinnitus were resolved (1/1) and aural fullness disappeared in 87.5% of ears (7/8). Nasal obstruction (2/2) and postnasal drip (2/2) were improved after surgery.* Conclusions*. Based on this preliminary report of a case series in a limited sample size, we suggest that using the transoral PEAK PlasmaBlade for adenoidectomy guided by video nasoendoscopy is a safe and feasible surgical technique, allowing remarkable outcomes by providing precise tissue removal, effective hemostasis, and painless postoperative recovery.

## 1. Introduction

Adenoid hypertrophy indicates the unusual growth of the adenoid. In chronic infection, the adenoid pad can remain enlarged for a long time, even in adulthood. Subsequent adverse consequences, including nasal obstruction, snoring, mouth breathing, aural fullness, and otitis media with effusion (OME), may result. In pediatric patients, enlarged adenoid that causes inability to breathe through the nose—resulting in chronic mouth breathing—can further lead to abnormalities in dental and facial growth. Proper treatment of adenoid hypertrophy often involves surgical removal.

The traditional surgical technique, which is performed blindly or under the view of laryngeal mirror using an adenoid curette or adenotome, has been challenged for inadequate reduction of hypertrophic adenoid tissue, especially in situations of involvement over the intranasal region, nasopharyngeal roof, and peritubal and retrotubal (pharyngeal recess) regions [1–5]. Methods of adenoidectomy have evolved in the past two decades following the introduction of the endoscope and powered surgical instruments. Under the direct view of endoscopes, either transorally or transnasally, techniques such as the conventional curette, Blakesley forceps, suction diathermy, microdebrider, and coblation can be applied to give a more appropriate alternative for adenoidectomy [1, 6–8].

A new low-thermal-injury electrosurgical device, the pulsed electron avalanche knife (PEAK), which is capable of the simultaneous division of tissue and coagulation of blood vessels, has been widely used in ear, nose, and throat (ENT) procedures such as tonsillectomy and adenoidectomy. In this paper, we report our experience of adenoidectomy via a transoral PEAK PlasmaBlade under the direct view of video nasoendoscopy. Our surgical techniques, their advantages and disadvantages, and significant outcomes are discussed.

## 2. Materials and Methods

### 2.1. Study Approval and Patients

This study was approved by the Institutional Review Board of the Tri-Service General Hospital, National Defense Medical Center, Taipei, Taiwan (Protocol number 1-104-05-137). Between April 2013 and May 2015, six patients (five male and one female) with adenoid hypertrophy were surgically treated using a PEAK PlasmaBlade by authors CYK and CHW. These patients (aged 6–25 years) presented with swollen adenoids at different grades that came into contact with the torus tubarius or even impeded the visualization of the tube ostium with ipsilateral or bilateral symptoms like middle ear effusion, aural fullness, tinnitus, snoring, and postnasal drip. Patients who displayed symptoms of allergic rhinitis had already been treated for a minimum of 8 weeks with nasal corticosteroid sprays. The preoperative information is summarized in Table 1.

Before and after surgery, all patients underwent nasal endoscopy to define the grading of hypertrophic adenoids (Table 1) and postoperative outcome (Table 2). A schematic diagram of a modified grading scale for estimating hypertrophic adenoid size according to the proposed adenoid staging system was shown by Parikh et al. [9]. Pure tone audiometry (PTA), tympanometry, and Eustachian tube functional tests were carried out before and after surgery to assess middle and inner ear functions. To assess patients' postoperative pain experience and the status of acute pain management, the pain ratings were obtained within 24 hours of surgery using the Wong-Baker Faces Pain Rating scale (0 = no pain; 5 = worst pain) [10].

The mean follow-up period was 23.8 months, with a range of 16 to 35 months. All patients were asked to report whether their symptoms had deteriorated, remained unchanged, improved, or were totally relieved at 6 months postoperatively and the latest follow-up visit.

### 2.2. Surgical Technique

Patients were lying in the low Fowler's position (15–30 degrees) with their head held in neutral position under general anesthesia, in the same manner as the setting for endoscopic sinus surgery. The nasal cavity was first packed with decongestant (0.05% oxymetazoline hydrochloride) soaked patties 10 min before the start of surgery. Transoral exposure was obtained using a Dingman mouth gag. To improve access to the nasopharynx, a 14-French red rubber Robinson catheter could be placed through either side of nasal passage and brought out of the mouth to provide soft palate retraction. The adenoids were inspected by introducing a 0° rigid endoscope (Karl Storz Hopkins II 7230AA), 4 mm in diameter and 18 cm in length, into the nostril. A 3-chip-CCD-Camera was mounted at the endoscope and a high resolution video monitor was used.

**Figure 1:**
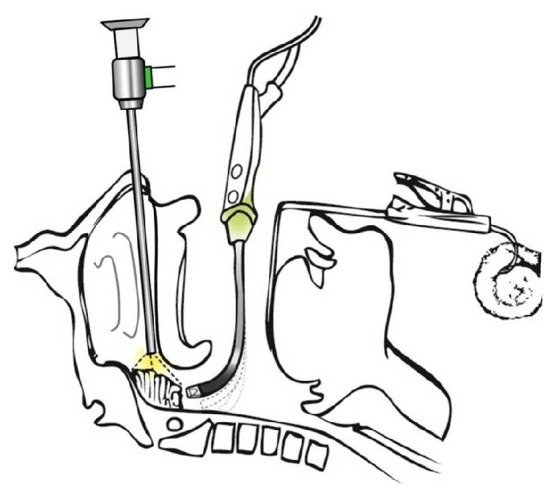
Schematic illustration of the patient and instrument setting.

**Figure 2:**
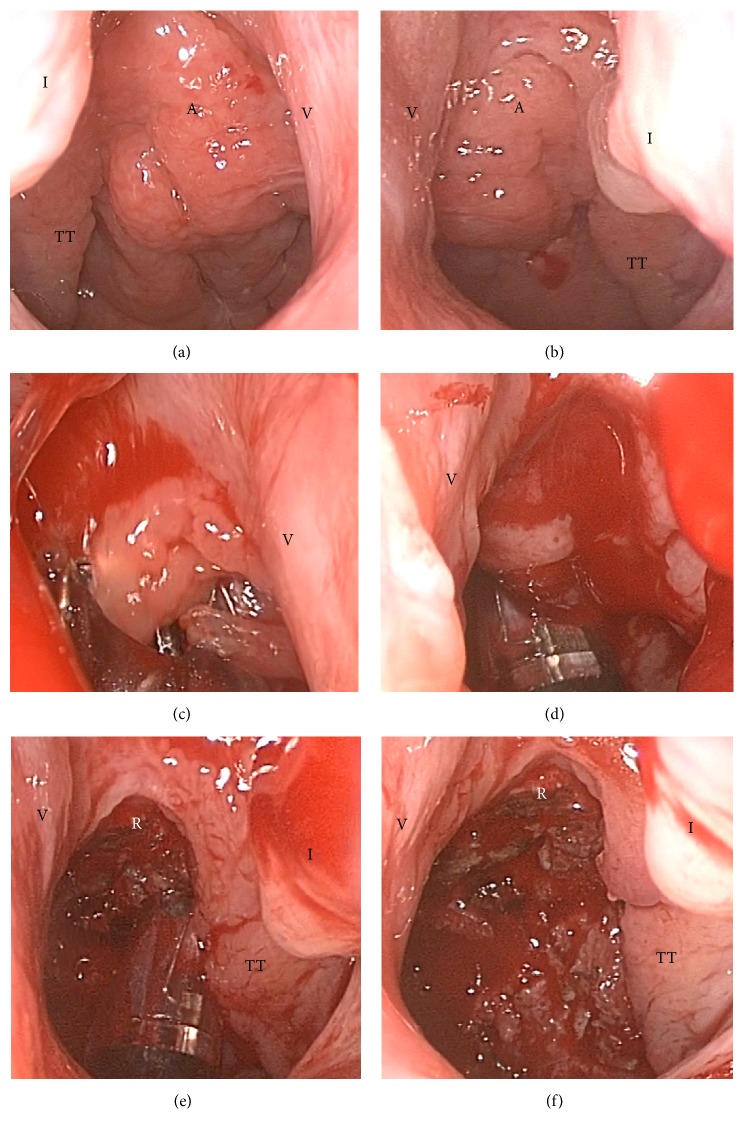
Sequential intraoperative steps of nasoendoscopic adenoidectomy using the transoral PlasmaBlade from Case 6. (a) Display of adenoid before surgery via the right nasal passage. (b) Endoscopic view via the left nasal passage. (c) The PlasmaBlade reaches the upper border of the choana. (d) Removal of the central bulk of the adenoid. (e) Reduction of peritubal and retrotubal adenoid tissues. (f) Postoperative view via the left nasal passage. A = adenoid; I = inferior turbinate; R = roof of nasopharynx; TT = torus tubarius; V = vomer.

The PEAK PlasmaBlade (Medtronic Inc., Minneapolis, MN, USA) was introduced through the mouth into the nasopharynx and choanal opening (Figure 1). With its flexible bending property, not only can the tip of the PlasmaBlade reach the central bulk of the adenoid, but it can also access the choana, the roof of the nasopharynx, and the peritubal and even retrotubal regions, guided by video nasoendoscopy. Adenoidectomy was begun from the upper border of choana, followed by sequential removal of adenoid tissues around the Eustachian tube opening, including the peritubal and retrotubal areas, and the nasal floor (Figure 2). Care should be taken to preserve the velopharyngeal function by leaving a rim of adenoid tissue over the Passavant ridge. To prevent damage, the mucosa covering the torus tubarius and the structure of the tubaric ostium should be clearly identified. Intraoperative tissue resection and hemostasis allow for switching between cutting and coagulation modes on the handpiece. There is no need to use 3% hydrogen peroxide as a hemostatic agent packing during or at the end of surgery. According to the guideline and regulation of the diagnosis-related group (DRG) payments on health-care in Taiwan, in this study, all adenoidectomy specimens were sent for pathological examination.

## 3. Results

### 3.1. Case 1

A 23-year-old man was referred and presented with complaints of nasal blockage, as well as bilateral ear fullness since childhood. He had been diagnosed with OME and had twice undergone myringotomy with tympanostomy tube placements. The symptom of ear fullness recurred following tube extrusion and the stuffy nose was always there. Nasoendoscopy revealed large adenoid blocking the Eustachian tubes and nasal passage and abutting the vomer, which was characterized as Grade 3 adenoid hypertrophy. Otoscopy showed fluid accumulating in the bilateral middle ears and accompanied by conductive hearing loss with an air-bone gap (ABG) of 25 dB in the left ear and 30 dB in the right ear.

Adenoidectomy with the PEAK PlasmaBlade and myringotomy were arranged. At the 24-month follow-up visit, closure of the ABG was maintained. Tympanometry revealed Type A in the right ear and Type C in the left ear, where aural fullness remained present although the patient was able to ignore it.

### 3.2. Case 2

An 8-year-old girl exhibiting microtia on the left side was referred with a 2-year history of intermittent snoring, as well as two sets of tympanostomy tube placements for chronic OME and conductive hearing loss of duration of 3 or more years. The mother noted that her child often kept her mouth open, especially during sleep. Physical examination of the oropharynx revealed relatively normal-size tonsils. However, nasoendoscopy showed enlarged adenoids obstructing the choanae with thick secretions accumulating in the nose, compatible with Grade 4 adenoid hypertrophy.

The patient's left ear represented a Type C microtia (Schuknecht's system of classification) [11] with undeveloped ear remnants and no ear canal. Otoscopy confirmed OME in the right ear. In May 2013, this patient had received adenoidectomy by PEAK PlasmaBlade to remove adenoidal tissue that blocked the torus tubarius. Concurrent myringotomy was carried out to remove middle ear effusion. Preoperatively, the PTA showed an average 20 dB ABG in the right ear. At the latest follow-up 12 months postoperatively, pure tone audiogram revealed normal hearing with closure of the ABG. There was no relapse of snoring or OME 24 months after surgery.

### 3.3. Case 3

The patient was a 6-year-old boy and presented with a 2-year history of recurrent OME of the left ear. He had received bilateral myringotomy with tympanostomy tube insertions 2 years earlier, followed by an additional tube insertion surgery over the left ear 1 year later. At out-patient department, otoscopy showed that the right tube had remained in place for 2 years, whereas the left tube had spontaneously extruded again with fluid collected behind an intact eardrum. Nasoendoscopic examinations revealed asymmetric adenoid hypertrophy, which was more prominent on the left side (Grade 4) but relatively smaller on the right side (Grade 3). Adenoidectomy was subsequently performed in conjunction with the insertion of a tympanostomy tube in the left ear. Within the 22-month follow-up period, the tube had fallen out and the patient was free of any ear symptoms. In addition, a noticeable improvement of snoring was reported by his mother.

### 3.4. Case 4

**Figure 3:**
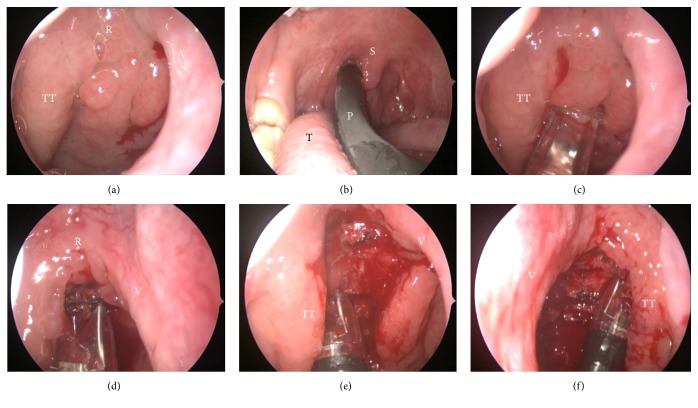
Intraoperative endoscopic views demonstrating the advantage of the PEAK PlasmaBlade used for adenoidectomy. These representative photos were captured from Case 4. (a) A Grade 3 adenoid hypertrophy is shown via the right nasal passage. (b) Transoral adenoidectomy with the PEAK PlasmaBlade. (c) Blade tip working on the right peritubal area. (d) Manipulating the blade tip toward the roof of the nasopharynx and (e) toward the retrotubal area of the right Eustachian tube. (f) Rotating the blade tip toward the retrotubal area of the left Eustachian tube. P = PlasmaBlade; R = roof of nasopharynx; S = soft palate; T = tongue; TT = torus tubarius; V = vomer.

**Figure 4:**
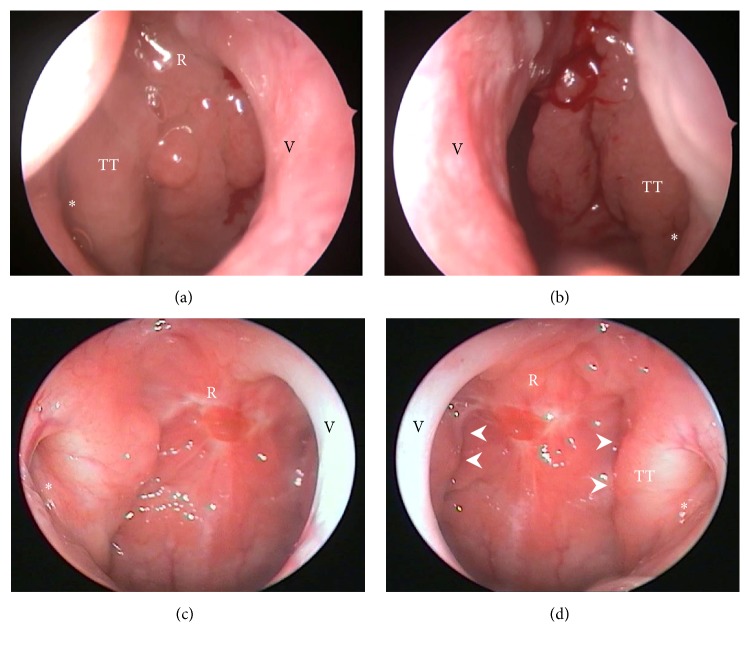
Postoperative outcome from Case 4. (a) Prior to and (b) during surgery, there were hypertrophic adenoid abuts on the torus tubarius which resulted in narrowing the right and left Eustachian tube opening (asterisk) as a slit-like appearance. (c, d) At 6-month postoperative follow-up, there is a significant reduction of adenoid volume around the roof of nasopharynx and the peritubal and retrotubal regions by showing a clear division between the adenoid pad and torus tubarius (arrow heads, panel (d)). A more dilated opening of the Eustachian tube is observed postoperatively (asterisk, panels (c) and (d)). R = roof of nasopharynx; TT = torus tubarius; V = vomer.

A 21-year-old man presented to our clinic with a 2-month history of hearing impairment in the right ear. He also reported having suffered from nasal obstruction and intermittent nasal discharge for many years. Nasoendoscopy showed bilateral inferior turbinate hypertrophy; in addition, a Grade 3 swollen adenoid that blocked the Eustachian tube opening and partially occluded the bilateral choanae was also noted (Figure 3(a)). Unilateral conductive hearing loss due to OME was confirmed by hearing tests and otoscopy. Prior to adenoidectomy, an office procedure of tympanocentesis had been performed plus corticosteroid nasal spray treatment for 2 months, but the symptoms persisted. Endoscopic examination at the 6-month postoperative follow-up showed near complete removal of hypertrophic adenoid tissues (Figures 4(c) and 4(d)). Functional recovery of the Eustachian tubes and adequate ventilation of the middle ear spaces were achieved, shown as Type A in a normal tympanogram.

### 3.5. Case 5

A 25-year-old man was referred with complaints of aural fullness and tinnitus in the left ear for months. Oral antihistamines and corticosteroids nasal spray were prescribed by the primary care physician to treat allergic disease-related Eustachian tube dysfunction. After treatment for 2 months, symptoms of tinnitus had become intermittent but not completely subsided, and the sensation of aural fullness had persisted, although pure tone audiometry showed normal hearing thresholds. Normal middle ear function bilaterally was also characterized by a Type A tympanogram, normal static immittance, and normal crossed and uncrossed reflex thresholds.

Nasoendoscopic examinations revealed Grade 2 adenoid hypertrophy abutting the torus tubarius, and this could have been contributing to the patient's symptoms. The patient decided to have adenoidectomy surgery. Postoperatively, the patient gradually lost the sensation of aural fullness and tinnitus, and he remained free from the above symptoms over the next 12 months.

### 3.6. Case 6

The patient was a 25-year-old man and presented with a history of prior septomeatoplasty to treat nasal obstruction 2 years earlier. However, the patient remained symptomatic despite surgical treatment. Furthermore, postnasal drip and aural fullness were also noted. Hearing tests including PTA and tympanometry showed normal results. Nasoendoscopic examinations revealed a widely patent meatus with partially resected inferior turbinates. With the scope moving forward, a huge hypertrophic adenoid was found to block the choanae, and this was characterized as Grade 4 adenoid hypertrophy. After adenoidectomy with the PEAK PlasmaBlade, the patient reported significant relief of his nasal obstruction. Aural fullness subsided, and the patient remained free from this symptom over a 10-month follow-up period.

## 4. Discussion

Although symmetric enlargement of adenoid tissues usually causes bilateral symptoms, unilateral symptoms resulting from hypertrophic adenoids cannot be ignored, as shown in Cases 4 and 5. In case of asymmetrical adenoid enlargements and resultant ipsilateral symptoms, as shown in Case 6, thorough history taking and physical examination are mandatory. Although we sent all of the adenoidectomy specimens for pathological analysis and the report showed no malignancy, histopathological screening of tonsillectomy and/or adenoidectomy (T&A) specimens without preoperative risk factors is not recommended [12, 13]. Instead, gross pathological evaluation of routine T&A specimens by a pathologist may be sufficient [13].

The sensations of aural fullness and tinnitus are frequently observed in the diagnosis of Eustachian tube dysfunction. However, both sensations may not be accurately reflected by tympanometry examination, as shown in our Case 5, suggesting that there is still a gap in interpreting the Eustachian tube function using indirect measurement of the compliance of the tympanic membrane and middle ear.

Although adenoid tissues would undergo regression toward the adolescent period, they may represent the chief cause of nasal obstruction in adults, as shown in previous research [14, 15] and our Case 6. Since the most common causes of nasal obstruction in adults are related to anatomic variations, such as deviated nasal septum and inferior turbinate hypertrophy, physicians should be aware of an uncommon condition resulting from adenoid hypertrophy.

With the evolution of endoscopic sinus surgery [2, 4, 5, 16, 17], the introduction of endoscopic vision during curettage adenoidectomy seems rational and optimal compared to use of the mirror for indirect vision. Several manipulations have been adopted to improve the adenoidectomy technique by introducing different surgical instruments from different routes under endoscopic vision, for example, transoral adenoidectomy guided by transoral or transnasal endoscope and transnasal adenoidectomy guided by transnasal or transoral endoscope [1].

The introduction of powered instrumentation has created more benefits for adenoidectomy. Microdebriders, which allow more precise and efficient removal of adenoid tissue, are widely used and well documented, although relatively limited maneuverability and difficulty in approaching the inferior nasopharynx had been reported [3, 18, 19]. Coblation technology uses bipolar radiofrequency energy to cause tissue dissolution at relatively low temperatures (between 40°C and 70°C) with simultaneous coagulation, thereby resulting in minimal intraoperative bleeding and rapid postoperative recovery after adenoidectomy [20, 21].

The PEAK PlasmaBlade is also a low-temperature device that uses pulsed radiofrequency to generate a plasma-mediated discharge along the exposed rim of an insulated blade and creates an effective cutting edge. It has been widely applied in the ENT field and is considered to provide scalpel-like cutting precision, electrosurgical-like hemostasis, less tissue injury, and minimal scar formation [22]. We found that a great advantage of the PEAK PlasmaBlade is that it offers a rotatable and bendable blade tip on a handpiece, thus making it easy to access the roof of nasopharynx and the peritubal and retrotubal regions (Figure 3). Such delicate approaches allow a significant reduction of hypertrophic adenoids which could be achieved and impact on subsequent functional recovery or even anatomic correction of the Eustachian tube opening (Figure 4).

The rates of residual adenoid tissue are relatively significant and conventional curettage adenoidectomy often misses a substantial volume of adenoid tissue. From a prospective study conducted on 99 patients undergoing adenoidectomy by blunt curettage, in 80% of the patients, a residue of lymphatic tissue was still present on the pharyngeal roof near the choanal opening or along the torus tubarius on either side of the nasopharynx [23]. By applying the PEAK PlasmaBlade in adenoidectomy, all six cases experienced almost total or near-total symptom relief after surgery without any complications. Postoperative pain intensity was estimated using the pain measurement scale and all 6 patients felt hurts just a little bit (scored 1). Regular analgesic medication is unnecessary even for pediatric patients.

We consider that a transnasal endoscopic view is the most recommended approach for otolaryngologists because it has the shortest learning curve by means of daily practice in nasoendoscopic manipulation encountered in office examination or sinus surgery in the operating room. Unlike conventional techniques that put all instruments in the same route, the transnasal endoscope is positioned superiorly to the surgical field and farther away from the surgical instruments; this facilitates the surgical process by preventing endoscope lens from being contaminated with blood staining and reducing clean times. Regarding the adenoidectomy route for introducing surgical instruments, we preferred the transoral route in view of a relatively large working space for manipulating instruments through the oropharynx. This makes nasoendoscope-assisted transoral approach also very suitable for children.

The cost of disposable instruments is often more expensive than traditional reusable ones. Nowadays use of disposable surgical instruments as standard procedures has become popular in many hospitals. Compared with traditional instruments for adenoidectomy, the PEAK PlasmaBlade costs an extra USD$ 500 in our country. Minimally invasive and delicate operations have been widely adopted, which give rise to surgeon's preference in new interventions as long as the price is affordable to the patient.

Despite the fact that study is limited by its small sample size and lack of a control group, we were encouraged to observe the effective impact on patients undergoing the interesting procedure.

## 5. Conclusions

Using a transoral PEAK PlasmaBlade for adenoidectomy guided by video nasoendoscopy was demonstrated to achieve superior clinical performance in terms of good visualization, complete and precise tissue removal, effective hemostasis, and painless postoperative recovery. This technique is suitable for patients with no age restriction and is highly recommended for pediatric patients with adenoid hypertrophy. Since our study is a preliminary report with a restricted number of patients, this topic deserves further observation with larger case numbers.

## Figures and Tables

**Table 1:** Preoperative information of patients.

Patient number	Sex/age (y)	Preoperative symptoms/complaints	Side (s)	Grading of adenoid hypertrophy	Preoperative tympanogram (s)
1	M/16	OME NO	Bilateral	Symmetric, Gr 3	Type B, bilateral
2	F/8	OME NO Snoring	Bilateral	Symmetric, Gr 4	Type B, right
3	M/6	OME NO Snoring	Bilateral	Asymmetric, Left: Gr 4 Right: Gr 3	Type B, bilateral
4	M/21	OME NO Postnasal drip	Right	Symmetric, Gr 3	Type A, left Type B, right
5	M/25	Aural fullness Tinnitus	Left Left	Symmetric, Gr 2	Type A, bilateral
6	M/21	Aural fullness NO Postnasal drip	Bilateral	Symmetric, Gr 4	Type A, bilateral

OME = otitis media with effusion; NO = nasal obstruction; Gr = Grade.

**Table 2:** Comparative symptoms/signs.

	Preoperative	Postoperative
Aural fullness (sides)	8	1
Tinnitus (sides)	1	Nil
Type B tympanogram (sides)	6	Nil
Conductive hearing loss (sides)	6	Nil
Nasal obstruction	5	Nil
Snoring	2	1
Postnasal drip	2	Nil
